# Impact of Polyallylamine Hydrochloride on Gene Expression and Karyotypic Stability of Multidrug Resistant Transformed Cells

**DOI:** 10.3390/cells9102332

**Published:** 2020-10-21

**Authors:** Larisa Alekseenko, Mariia Shilina, Irina Kozhukharova, Olga Lyublinskaya, Irina Fridlyanskaya, Nikolay Nikolsky, Tatiana Grinchuk

**Affiliations:** Department of Intracellular Signaling and Transport, Institute of Cytology, Russian Academy of Sciences, Tikhoretskay Ave 4, St. Petersburg 194064, Russia; shili-mariya@yandex.ru (M.S.); kojuxarova@mail.ru (I.K.); o.lyublinskaya@mail.ru (O.L.); irfreed@yahoo.com (I.F.); nik.n.nikolsky@gmail.com (N.N.); grintat@bk.ru (T.G.)

**Keywords:** polyallylamine hydrochloride, Chinese hamster cells V-79, multidrug resistance, *p53*, *c-fos*, *topo2-α*, *hsp90*, *hsc70*, karyotypic stability, oncogenic transformation, doxorubicin

## Abstract

The synthetic polymer, polyallylamine hydrochloride (PAA), is found in a variety of applications in biotechnology and medicine. It is used in gene and siRNA transfer, to form microcapsules for targeted drug delivery to damaged and tumor cells. Conventional chemotherapy often does not kill all cancer cells and leads to multidrug resistance (MDR). Until recently, studies of the effects of PAA on cells have mainly focused on their morphological and genetic characteristics immediately or several hours after exposure to the polymer. The properties of the cell progeny which survived the sublethal effects of PAA and resumed their proliferation, were not monitored. The present study demonstrated that treatment of immortalized Chinese hamster cells CHLV-79 RJK sensitive (RJK) and resistant (RJKEB) to ethidium bromide (EB) with cytotoxic doses of PAA, selected cells with increased karyotypic instability, were accompanied by changes in the expression of *p53* genes *c-fos*, *topo2-α*, *hsp90*, *hsc70*. These changes did not contribute to the progression of MDR, accompanied by the increased sensitivity of these cells to the toxic effects of doxorubicin (DOX). Our results showed that PAA does not increase the oncogenic potential of immortalized cells and confirmed that it can be used for intracellular drug delivery for anticancer therapy.

## 1. Introduction

Synthetic biopolymers with biodegradable and biocompatible properties are extensively used in the biomedical field, such as cancer treatment, gene therapy, stem cell research, development of cell scaffolds, and cell technologies for organ regeneration [1,2]. Water soluble synthetic polymeric cation polyallylamine hydrochloride (PAA), a representative of such polymeric carriers, has many biomedical applications. PAA is used in the formation of microcapsules for targeted delivery of drugs, multilayer films covering biomedical implants. It is implicated in gene and siRNA transfer due to electrostatic bonds between negatively charged phosphate groups of nucleic acids and positively charged polycationic groups [3,4,5]. In tissue engineering, the surface characteristics of a biomaterial are very important factors determining its biocompatibility. They affect the cell attachment and growth on the substrate. In many cases, the surface must be modified and designed in the right direction. Modification of non-adhesive surfaces with polyelectrolyte multilayer films has recently been proposed as a powerful method that promotes the growth of various cells [6,7,8].

Some researchers believe that PAA is a biologically neutral material [9]. However, an analysis of the genetic expression of human vascular smooth muscle cells 24 h after exposure to PAA microcapsules exhibited profound changes in gene expression related to cytoskeleton organization, cell cycle, cell adhesion, migration ability, and the mitogen-activated protein kinase signaling pathway (MAPK) [3]. Cytogenetic analysis of Chinese hamster cells CHLV-79 RJK (RJK) resistant to ethidium bromide (RJKEB) 24 h after the exposure to PAA revealed multidirectional destabilization of the karyotype (the occurrence of aneuploidy and the appearance of chromosomal aberrations) [10]. It was also demonstrated that adhesion of RJK to the surface coated with PAA varied depending on the PAA concentration in solution. PAA at concentration of 0.01 μg/mL enhanced cell attachment compared to cell adhesion to an untreated surface. An increase in PAA concentration led to dose-dependent inhibition of cell adhesion. Cell staining with trypan blue showed that concentration of PAA 100 μg/mL was sublethal [11]. The effect of PAA on the properties of cells that survived after treatment with sublethal doses of the biopolymer has not been studied and remains unclear.

The use of biopolymer carriers for drug delivery can be part of complex drug therapy in the treatment of cancer. In this regard, for the development of safe carriers that contain PAA, it is important to signify the whole spectrum of possible effects, including the delayed effects that PAA has on transformed cells and drug resistant cell populations. With any subcytoxic effects on the tumor, there is a risk of increasing its insensitivity to drugs and the development of the multidrug resistance (MDR). MDR is defined as the resistance of cells to the cytotoxic effect of structurally heterogeneous and functionally different chemotherapeutic agents and is considered as a main challenge in cancer treatment. A typical mechanism that ensures cell resistance to drugs is the expression of the transmembrane glycoprotein with the molecular weight of 130–200 kDA, called P-glycoprotein (“permeability” glycoprotein), product of the *mdr1* gene [12]. Functionally, P-glycoprotein, or MDR1, is an energy-dependent pump that removes drugs from resistant cells, thereby reducing their intracellular content. Enhanced MDR1 expression is characteristic of typical MDR tumors. In the case of atypical MDR, MDR1 expression remains unchanged, but it is possible that expression of DNA topoisomerases (e.g., topo-2α), ribozymes that play an important role in DNA replication, transcription and chromosome separation is altered [13]. The process of oncogenic transformation and MDR appearance is accompanied with changed expression of many genes. P53 tumor suppressor is a critical component of the system that maintains the genetic stability of animal and human cells. The suppression of this gene is observed in almost all types of human cancer. It facilitates the tumor cell to accumulate additional mutations and progress towards higher malignancy [14]. A key player in oncogenesis is also the *c-fos* proto-oncogene, which is involved in several cellular events, including cell proliferation, regulation of genes associated with hypoxia and angiogenesis [15]. Heat shock proteins (HSP), which are involved in various cell processes, such as protein folding, apoptosis, autophagy, and cellular immunity, are now considered to be related to oncogenesis as well. HSP protect cancer cells from environmental and pharmacological stress factors and can interfere with cancer therapy. Several studies have demonstrated the relationship between HSP and drug resistance, as well as the possibility of their use as biomarkers for detecting tumors [16].

The study of the MDR phenomenon in rodent and human cells in culture showed that these cells underwent a change in the permeability of the cell membrane, as well as genetic changes recorded both at the karyotypic and molecular levels. At the molecular level, this is associated with increased expression of *mdr1* gene. At the karyotypic level it is recorded as the appearance of an additional genetic material, homogenous stained regions (HSR) in one of the chromosomes, as well as double minute chromosomes (DM) considered as morphological manifestations of *mdr1* gene amplification [17,18], and markers of cells with MDR.

The studies of PAA effect on cells have been mainly focused on their morphological and genetic characteristics immediately or a few hours after exposure to the polymer [3,10]. The properties of cell progeny survived sublethal effects of PAA and resuming their proliferation have not been monitored. There is also no data on the effect of sublethal doses of PAA on cell populations with MDR. In this regard, the aim of this work was to investigate the gene expression and karyotypic stability of the descendants of Chinese hamster cells RJK with and without MDR that survived after exposure to PAA in sublethal concentration.

## 2. Materials and Methods

### 2.1. Cells

The study was performed on Chinese hamster fibroblasts RJK, RJKEB, RJK-PAA, and RJKEB-PAA cells. The constant transformed RJK (CHL V-79 RJK) cells were provided by Dr. F. Ruddle (Yale University, New Haven, CT, USA). RJKEB cells were obtained by Dr. Ignatova and Dr. Artsybasheva (Institute of Cytology RAS, Russia) using gradual selection of RJK cells for resistance to 5 μg/mL EB. RJK-PAA and RJKEB-PAA cells were progenies surviving after PAA exposure to RJK and RJKEB cells, respectively. The cells were maintained in Dulbecco’s modified Eagle’s medium (DMEM) /F12 medium (Gibco, Brooklyn, NY, USA) with 10% bovine fetal serum (HyClone, Logan, UT, USA), 1% antibiotic-antimycotic solution, and 1% GlutaMAX (Gibco, Brooklyn, NY, USA). The cells were subcultured 1:3 twice a week using 0.05% trypsin with ethylenediaminetetraacetic acid (EDTA) (Invitrogen, Waltham, MA, USA). The proliferation activity was assessed by creating cell growth curves. The average population doubling time was estimated using the formula: Td = tlg2/log (Nt/N0), with Td—the average population doubling time, t—the population growth time, Nt—the number of cells after time t, N0—the initial number of cells.

### 2.2. Treatment Cells with Polymer Polyallylamine

PAA was synthesized at the Institute of Macromolecular Compounds of RAS. PAA average M_w_ ~ 44,000 [19]. The concentration of PAA for cell treatment was 100 μg/mL. The polycation was added to the submonolayer cell culture in the complete culture medium. The exposure time with PAA was 1.5 h, after which the cells were washed twice with phosphate-buffered saline (PBS) (Sigma, St. Louis, MO, USA) and added growth medium.

### 2.3. G-banded Karyotyping

Colchicine (Merck, Germany), final concentration of 3.6 μg/mL, was added to the cell culture that reached a confluence of 80% for 1–1.5 h. at 37 °C. Then, the medium was removed; cells were detached with 0.05% trypsin (Biolot, Russia) and centrifuged (1000 rpm). The pellet was resuspended and treated with 0.075 M KCl for about 1 h. The cell suspension was centrifuged, the pellet was resuspended and cells were fixed by a mixture of methanol with acetic acid (3:1). The fixing solution was changed three times. The total fixation time was 1.5 h. The fixed cell suspension was dropped on cold and wet slides. The slides were air dried at room temperature for one week. Then, the chromosomes were G-banded with the Giemsa stain (Fluka, Newport News, VA, USA) after previous trypsinization. Metaphase plates with well-spread chromosomes were assayed under the microscope Ampleval (Zeiss, Germany) with magnifications of 20× and 100×. The chromosomes were identified according to the standard nomenclature [20]. The work was carried out at the population level. In each sample we analyzed no less than 30 metaphase plates. Cell karyotyping was performed at the 4th passage after exposure to PAA.

### 2.4. Cell Cycle Analysis

Cells were harvested with trypsin-EDTA solution and suspended in fresh medium. Then 200 µg/mL saponin (Fluka, New York, NY, USA), 250 µg/mL RNase A (Sigma, St. Louis, MO, USA, R4642), and 50 µg/mL propidium iodide (Sigma, St. Louis, MO, USA) were added to each sample tube. After incubation for 60 min at room temperature, samples were analyzed with a CytoFLEX flow cytometer (Beckman Coulter, Indianapolis, IN, USA; 488 nm laser). Mean fluorescence intensity from 10,000 cells was acquired. Cell cycle analysis was performed using CytExpert v. 2.0 software (Beckman Coulter, Indianapolis, IN, USA).

### 2.5. Viability Analysis

Cells were harvested with trypsin-EDTA solution and suspended in fresh medium. Then 50 µg/mL propidium iodide (Sigma, St. Louis, MO, USA) was added to each sample tube. After incubation for 5 min at room temperature, samples were analyzed with a CytoFLEX flow cytometer (Beckman Coulter, Indianapolis, IN, USA; 488 nm laser). Mean fluorescence intensity from 10,000 cells was acquired. Cells were gated by size and granularity using FSC/SSC dot plot. Cell debris was excluded from the analysis.

### 2.6. qRT-PCR Assay

To analyze gene expression, total RNA was isolated with RNesy Micro Kit (Qiagen, Germany) according to the manufacturer’s instructions. RNA was quantified in a NanoDrop ND-1000 Spectrophotometer (NanoDrop Technologies, Inc., Wilmington, DE, USA). cDNA was obtained by reverse transcription of 500 ng RNA using the RevertAid H Minus First Strand cDNA Synthesis Kit (Thermo Fisher Scientific, Vilnius, Lithuania) according to the manufacturer’s instructions. It was subsequently amplified with specific primers, using DreamTaq™ PCR Master Mix (Thermo Fisher Scientific, Lithuania) with CycloTemp amplificator. For qRT-PCR cDNA was amplified with specific primers, using EvaGreen^®^ dye (Biotium) and DreamTaq™ PCR Master Mix (Thermo Fisher Scientific, Lithuania) in the BioRad CFX-96 real time system (BioRad, CA, USA), according to the kit’s enclosed protocol. The volume of the reverse transcription polymerase chain (RT-PCR) reactions was 20 µL. Gene expression was calculated with BioRad CFX Manager 3.1 software. Expression of target genes was normalized to actin gene. Primers and reaction conditions are presented in Table 1. All amplification reactions were performed in triplicate. Experiments were repeated at least three times.

### 2.7. MTT Assay

Cells in the log-phase of growth were seeded in 96-multiwall plates in 10^4^ cells per well. The next day DOX (Teva, Netherland) at concentrations 0–50 μg/mL was added to the cell growth medium. After 24 h 20 μL MTT (Sigma-Aldrich, St. Louis, MO, USA) solution (5 mg/mL 3-[4,5-dimethylthiazol-2-yl]-2,5-diphenyl tetrazolium bromide in PBS) was added to the control and tested cells. Cells were incubated for 3 h at 37 °C and 5% CO_2_. Mitochondrial dehydrogenases of viable cells cleave the tetrazolium ring, yielding purple formazan crystals. The crystals were dissolved in acidified isopropanol (Sigma-Aldrich, St. Louis, MO, USA). The resulting solutions were spectrophotometricaly measured at 570 nm with a microplate scanning spectrophotometer (Pikon, Russia). The change in optical density was evaluated by comparison with that of control samples.

### 2.8. Colony Forming Assay

Cells in the log-phase of growth were treated with DOX at concentrations 0–100 μg/mL for 6 h at 37 °C and 5% CO_2_. Following treatment, single cell suspension containing 200 cells in 4 mL was seeded into 5 cm Petri dishes (3 replicates). The incubation time for colony formation varied from 2 weeks for RJK, RJKEB cells and 3 weeks for RJK-PAA, RJKEB-PAA cells. Dishes were washed with PBS, fixed with 10% neutral buffered formalin solution for 15–30 min and stained with 0.01% (*w*/*v*) crystal violet (Sigma-Aldrich, St. Louis, MO, USA) in H_2_O for 30–60 min. Colonies containing more than 50 cells were counted using an inverted microscope (Zeiss, Germany). Cloning efficiency was estimated as the number of grown colonies/number of plated cells (%).

### 2.9. Statistical Analysis

Experiments were performed in triplicate. Excel and GraphPad PRISM 5.02 were used as statistical software. The results are expressed as mean ± SD. The Student’s *t*-test was used to determine the statistical significance of differences between two groups (cell growth, cell viability). One-way ANOVA with post-hoc Tukey HSD Test was used to determine the significance of differences among groups (gene expression, cloning efficiency). The null hypothesis was rejected at the 0.05 level of significance.

## 3. Results

### 3.1. Characteristics of Cell Lines RJK and RJKEB

**Figure 1:**
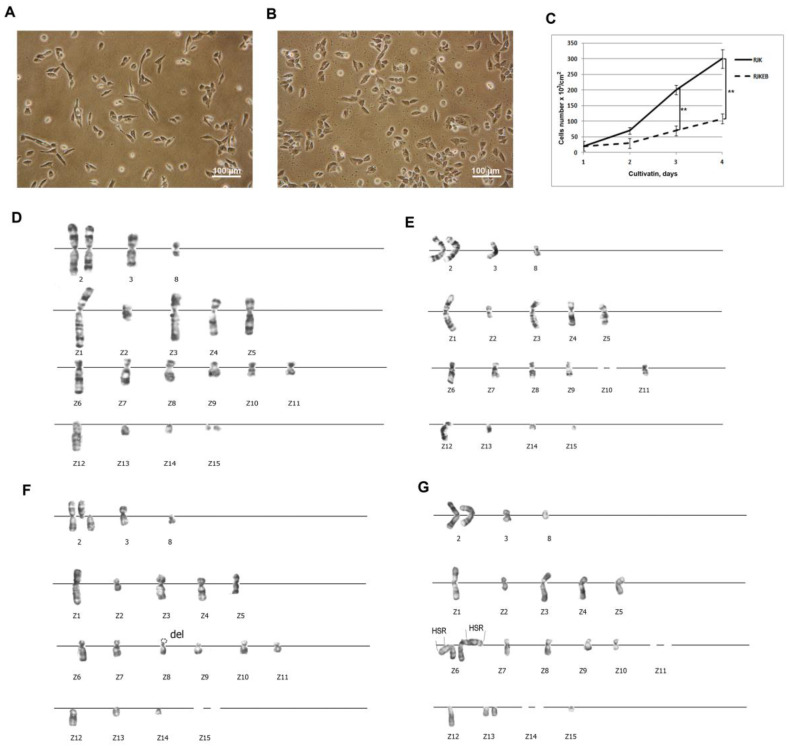
Characteristics of RJK and RJKEB cells. (**A**) RJK cells, live imaging, Ob: 10×; (**B**) RJKEB cells, live imaging, Ob: 10×; (**C**) RJK and RJKEB growth curves. To plot cell growth curves, the number of RJK and RJKEB cells was counted using FACS analysis for 4 days. The mean ± SD of three independent experiments (** *p* ˂ 0.01); (**D**) standard RJK karyotype. 2, 3, 8—normal chromosomes. Z1-Z15—marker chromosomes. n = 20; (**E**) RJK karyotype with loss of chromosome Z10, n = 18; (**F**) karyotype with centromeric break in chromosome 2, del p terZ8, chromosome Z15 is absent. n = 18; (**G**) karyotype of RJKEB cells. A cell variant with two copies of the Z6 chromosome labeled with HSR of different lengths. Chromosomes Z11 and Z15 are absent. n = 18. Ob: 100×. Abbreviations: RJK—Chinese hamster cells CHLV-79 RJK; RJKEB—RJK resistant to ethidium bromide; FACS—fluorescence-actevated cell sorting; del—deletion; p—short chromosome arm; q—long chromosome arm; n—number of chromosomes in the karyotype; HSR—homogeneously stained region.

Chinese hamster transformed fibroblast RJK cells and its subline RJKEB resistant to 5 μg/mL EB were used in this study. Since EB is a substrate for the majority of cell multidrug efflux pumps, EB-resistant cells were exploited as an example of cells with MDR phenotype. RJK cells have fibroblast-like morphology (Figure 1A). The population doubling time is 23 h. The density of the confluent monolayer is 300,000 cells/cm^2^ (Figure 1C). RJK cell karyotype was characterized by the presence of four normal Chinese hamster chromosomes (two copies of chromosome 2, one copy of chromosomes 3 and 8) and 15 rearranged, marker chromosomes (Z1–Z15) (Figure 1D). The modal chromosome number was represented by 17–19 chromosomes (Table 2). The population was characterized by increased structural instability of the chromosome 2 and variable occurrence of chromosomes Z2, Z14, Z15. Other changes were rare and random (Figure 1F).

RJKEB cells also have a fibroblast-like morphology, however, foci of polygonal cells are also found in the culture (Figure 1B). The population doubling time is 26 h. The density of the confluent monolayer is 120,000 cells/cm^2^ (Figure 1C). The karyotypic characteristics of this line (number of chromosomes, modal chromosome number and types of instability) did not differ from the cells of the original RJK line (Figure 1F). The variability of the number of chromosomal copies from cell to cell is from 0 to 3 (Table 2). The karyotypic marker of these cells was the presence of additional genetic material in the form of HSR at the locus 1q26 of chromosome Z6, a derivative of chromosome 1, at the location of the wild-type *mdr1* gene (Figure 1G). The length of HSR varied from cell to cell, which affected the chromosome index and the chromosome topology that they marked. Three types of HSR were detected in the analyzed cells. As a result, the length ratio of the short (p) and long (q) arms in different cells was different: p < q, p = q, p > q.

### 3.2. Morphology and Proliferative Features of RJK and RJKEB Cells after Exposure to PAA

**Figure 2:**
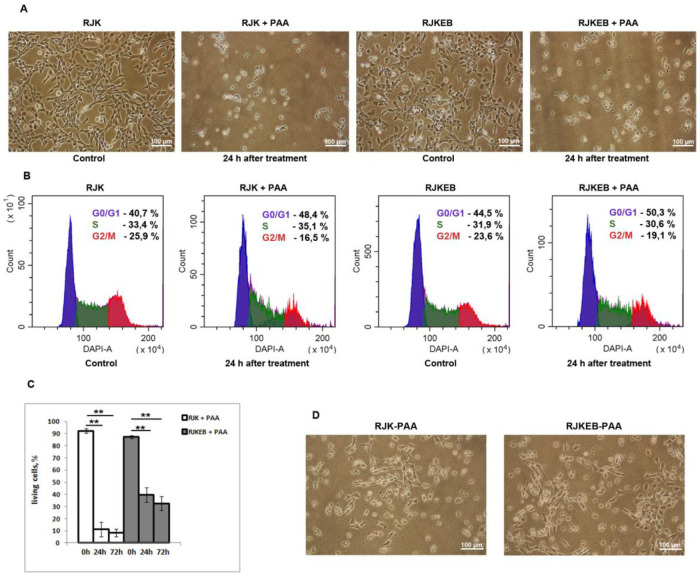
Analysis of RJK, and RJKEB cell populations after exposure to PAA. (**A**) Morphology of RJK and RJKEB cells 24 h after treatment with PAA. Ob. 10×. (**B**) FACS analysis of the cell cycle 24 h after PAA treatment. (**C**) FACS analysis cell viability 24 after PAA treatment. (**D**) Morphology of RJK and RJKEB cells resumed proliferation after PAA treatment. The mean ± SD of three independent experiments are presented (** *p* ˂ 0.01). Abbreviations: RJK—Chinese hamster cells CHLV-79 RJK; RJKEB—RJK resistant to ethidium bromide; PAA—polyallylamine hydrochloride, RJK-PAA—RJK cells after PAA treatment; RJKEB-PAA—RJKEB cells after PAA treatment; EB—ethidium bromide; FACS—fluorescence-actevated cell sorting.

To study the effect of the polymer on cell cultures, we chose PAA concentration of 100 μg/mL. It is sublethal for the RJK line and kills most cells [11]; 24 h after PAA treatment, RJK and RJKEB cells changed their morphology (Figure 2A). Most cells lost their fibroblast-like shape and were rounded. Flow cytometry assay demonstrated multiphase cell cycle arrest in RJKEB cells and accumulation RJK cells in late G1 and early S phase (Figure 2B). The viability of RJK and RJKEB cells exposed to PAA for 24 h decreased to 10% and 40% and after 72 h to 8% and 32%, respectively (Figure 2C). Within 72 h after treatment, the cell number in cultures of both lines did not increase however, later the cells resumed proliferation, acquired typical fibroblast morphology, gradually reached a monolayer and could be subcultured (Figure 2D).

### 3.3. Increased Karyotypic Instability of RJK and RJKEB Cells Survived after Exposure to PAA

**Figure 3:**
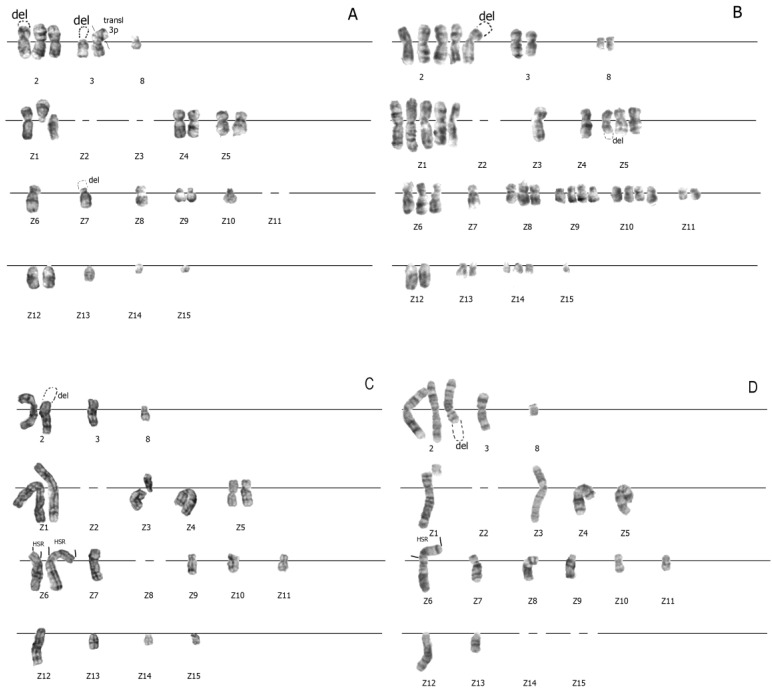
Changes in the karyotype of RJK and RJKEB cells survived after exposure to PAA. (**A**) Aneuploid RJK-PAA karyotype with 3 copies of chromosome 2; chromosomes—3, Z1, Z4, Z5, Z9, Z12—2 copies; lack of chromosomes Z2, Z3, Z11; del 2p ter (in one copy) and del Z7 pter (in one copy), del 3p (in one copy, tr 3p: 3p ter (in another copy). n = 23. (**B**) Aneupolyploid RJK-PAA karyotype, chromosome Z2 is missing; centromeric breaks in one copy of chromosome Z1 (p-arm, q-arm, in one copy); del 2p ter (in one copy) and delZ5qter (in two copies). n = 44. (**C**) Aneuploid RJKEB-PAA karyotype with impaired copying of chromosomes Z1, Z5, Z6; lack of chromosomes Z2, Z8; centromeric breaks in Z3 chromosome (p-arm, q-arm), the presence of HSR of different lengths in Z6. n = 20. (**D**) Aneuploid RJKEB-PAA karyotype with impaired copy number of chromosome 2 (3 copies), lack of chromosomes Z2, Z14, Z15; del 2q—ter (in one copy), break in Z1 pter. n = 17. Abbreviations: RJK—Chinese hamster cells CHLV-79 RJK; RJKEB—RJK resistant to ethidium bromide; PAA—polyallylamine hydrochloride; RJK-PAA—RJK cells after PAA treatment; RJKEB-PAA—RJKEB cells after PAA treatment; EB—ethidium bromide; del-deletion; ter-terminal end of the chromosome; p—short arm of the chromosome; q—long arm of the chromosome; n—number of chromosomes in the karyotype; HSR—homogeneously stained region.

Cell exposure to drugs or factors of endogenous or exogenous stress can lead to changed chromosome structures. In this regard, the next task of our study was a comparative analysis of the karyotype structure in descendants of RJK (RJK-PAA) and RJKEB (RJKEB-PAA) cells after PAA treatment. Karyotypic analysis of the chromosome structure in RJK-PAA and RJKEB-PAA cells was performed at the 4th passage of cultivation under standard conditions. It was found that in RJK-PAA cells, the variation in chromosome number increased compared to the control, while the modal class was not distinctive. The number of chromosomal copies within the karyotype increased and varied from 0 to 5 (Table 2). The frequency of cell occurrence with morphological abnormalities in the chromosome structure compared to control cells not treated with PAA increased twice. Breaks in chromosomes 3, Z1, Z4, Z7, Z8 were not random (Figure 3A,B). Up to three rearranged chromosomes could be present within the karyotype, while in the control population there was no more than one rearrangement per karyotype. The karyotype of RJKEB-PAA cells similar to RJK-PAA karyotype was unstable (Figure 3C,D). The modal class of chromosome numbers was well defined but differed from the modal chromosome number in RJKEB cells (Table 1). The number of chromosome copies varied in cells from 0 to 3 (Table 1). The number of chromosomes involved in the rearrangement did not differ significantly from RJK-PAA cells. Chromosomes 2, Z1, Z3, Z6, Z8, and Z13 (Figure 3C) frequently participated in the rearrangements. Two chromosomes (Z1, Z8) were involved in rearrangements both in RJK-PAA cells and RJKEB-PAA cells. Rearrangements in chromosome Z1 occurred more often than in Z8 (Figure 3A,D). Cellular variants with HSR, where p-arm is >q-arm were absent in RJKEB-PAA cells. These data show that PAA treatment of Chinese hamster RJK cells regardless of the level of their multidrug resistance altered their genetic status and significantly changed stability of the genetic apparatus at the karyotype level.

### 3.4. Expression of Genes Involved in Oncogenic Transformation in Progeny of RJK RJKEB Survived after Exposure to PAA

**Figure 4:**
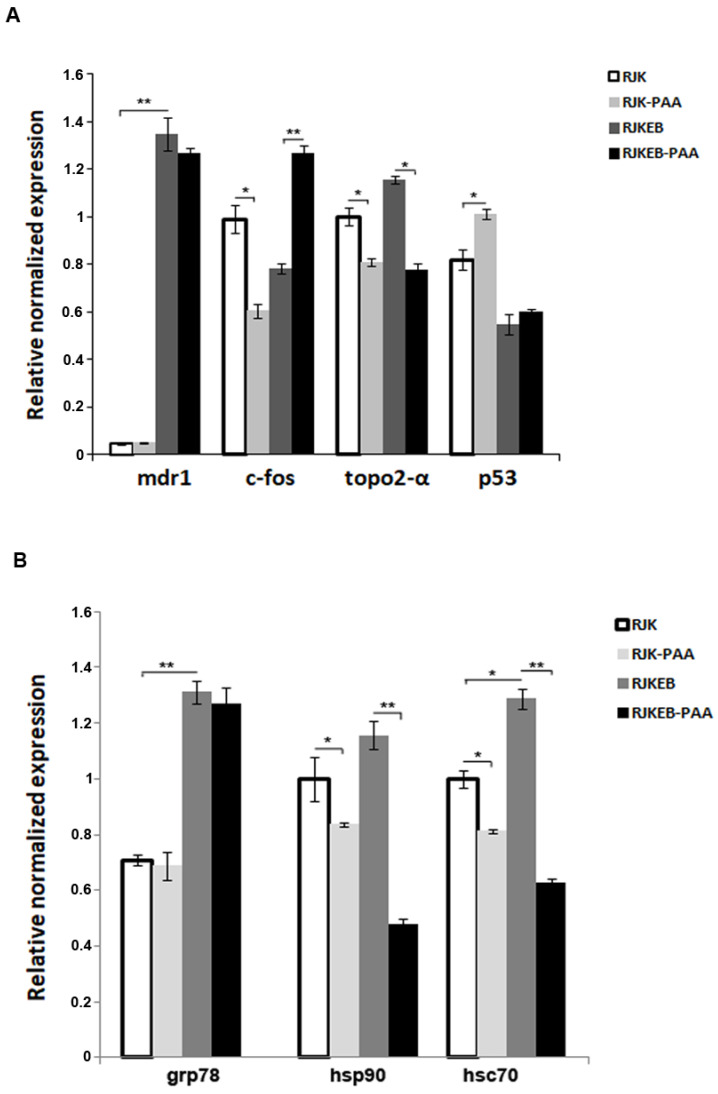
Gene expression on RJK and RJKEB cells survived after PAA exposure. (**A**) qRT-PCR analysis of the gene *mdr1*, *c-fos*, *topo2-α*, *p53* level in RJK, RJK-PAA, RJKEB, RJKEB-PAA cells. (**B**) qRT-PCR analysis of the gene *grp78*, *hsp90*, *hsc70* level in RJK, RJK-PAA, RJKEB, RJKEB-PAA cells. The mean ± SD of three independent experiments are presented (* *p* < 0.05; ** *p* ˂ 0.01). Abbreviations: RJK—Chinese hamster cells CHLV-79 RJK; RJKEB—RJK resistant to ethidium bromide; PAA—polyallylamine hydrochloride; RJK-PAA—RJK cells after PAA treatment; RJKEB-PAA—RJKEB cells after PAA treatment; EB—ethidium bromide; qRT-PCR—quantitative reverse transcription polymerase chain reaction.

Increased karyotypic instability of cell lines may be accompanied by a change in gene expression. Since the object of our study was cells with the MDR phenotype, we first analyzed the basal level of *mdr1* gene expression in RJK and RJKEB cells, and then in their progeny surviving PAA exposure. Real-time PCR (Figure 4) revealed that basal expression of the *mdr1* gene was 10 times higher in the RJKEB than in RJK cells, which correlates with the presence of HSR on chromosome Z6 in RJKEB cells. RJK and RJKEB cell progeny survived after exposure to a sublethal dose of PAA did not exhibit an increased expression of *mdr1* in comparison with the control cells. Besides, we analyzed the impact of PAA on the expression of several genes involved in oncogenesis. The basal expression level of proto-oncogene *c-fos*, which is known to participate in the regulation of *mdr1* gene expression and is overexpressed in various types of cancer, was higher in the RJK cells compared to RJKEB cells. Its expression significantly increased in the RJKEB cells after exposure to PAA unlike in EB-sensitive RJK cells. The expression of the key tumor suppressor gene *p53* enhanced in RJK-PAA but not in RJKEB-PAA cells compared to parent cells. The expression of *topo2-α* gene, which is associated with atypical multidrug resistance, decreased after exposure to PAA in both cell lines. Finally, we examined the influence of PAA on the expression of the molecular chaperon genes important for the proteostasis, stress-defense and viability of cancer cells. The basal level of *hsc70* and *grp78* was significantly higher in the RJKEB cells in comparison the RJK cells, whilst no significant differences in *hsp90* expression were found. Decreased expression of *hsp90* and *hsc70* was observed in cells after PAA exposure. The level of *grp78* remained unchanged in both cell lines.

### 3.5. PAA Reduced RJKEB Cell Resistance to DOX

**Figure 5:**
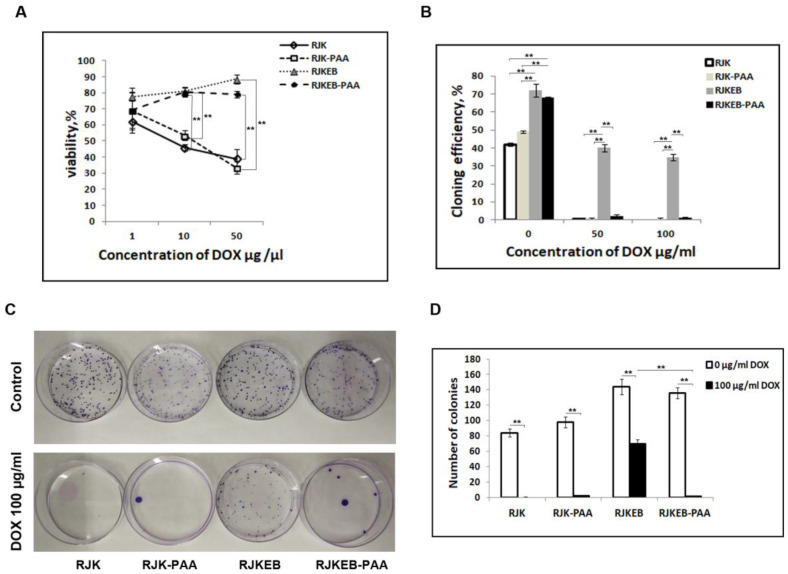
Viability of RJK, RJK-PAA, RJKEB, RJKEB-PAA cells after treatment with various doses of DOX. (**A**) Viability of RJK, RJK-PAA, RJKEB and RJKEB-PAA treated with DOX for 24 h. MTT assay. (**B**) The cloning efficiency of RJK, RJK-PAA, RJKEB, RJKEB-PAA after DOX treatment. (**C**) Petri dishes with colonies of RJK, RJK-PAA, RJKEB, RJKEB-PAA cells stained with crystal violet dye. (**D**) Quantitation of colonies in RJK, RJK-PAA, RJKEB, RJKEB-PAA cultures after DOX treatment. The mean ± SD of three independent experiments are presented (** *p* ˂ 0.01). Abbreviations: RJK—Chinese hamster cells CHLV-79 RJK; RJKEB—RJK resistant to ethidium bromide; PAA—polyallylamine hydrochloride; RJK-PAA—RJK cells after PAA treatment; RJKEB-PAA—RJKEB cells after PAA treatment; EB—ethidium bromide; DOX—doxorubicin.

Decreased expression of HSP and topo2-α may correlate with increased sensitivity of cancer cells to the chemical agents used in cancer therapy. We decided to test the resistance of cells that survived sublethal exposure to PAA to one of the MDR agents, DOX. Cells treated and untreated with PAA at passage 4 after restored proliferation were subjected to DOX. Cells were grown to submonolayer and treated with 1, 10, and 50 μg/mL DOX in the growth medium for 24 h. The cytotoxic effect of DOX was evaluated using the MTT test. The results of cell survival after treatment with DOX are presented in Figure 5. It can be seen that survival of RJK and RJK-PAA cells decreased from 70 to 30% with the increase in DOX dose (Figure 5A). The viability of the RJKEB cells both treated and untreated with PAA did not significantly decrease with DOX concentrations ranged from 1 to 10 μg/mL. After treatment with DOX at concentration of 50 μg/mL, the survival rate of the RJKEB-PAA line was slightly lower than RJKEB (*p* < 0.05). The original RJK line was sensitive to DOX, and the RJKEB line was resistant to DOX at concentrations of 1 to 10 μg/mL.

Cells of all four cell lines were cloned after 6-h exposure to 0, 50, and 100 μg/mL DOX. The results are presented in Figure 5B. The cloning efficiency of control untreated cells sensitive to EB was about 40–50% while it was 75–65% in lines resistant to EB. DOX treatment reduced the cloning efficiency of RJKEB cells to 35–40% (Figure 5B). The RJK line had no clones after DOX treatment. Exposure to PAA reduced the cloning efficiency to 1–2% in the resistant cell lines (Figure 5C). Thus, in our experiments PAA pretreatment affected the multidrug resistance of transformed cells and increased their sensitivity to DOX.

## 4. Discussion

Cancer remains a disease difficult to treat due to poor drug potency and side effects during the cancer therapy. Conventional chemotherapy is often unsuccessful in killing all cancer cells that leads to cross-resistance to a variety of other chemotherapeutics, so called MDR [21]. Synthetic polymers are trendy drug delivery platforms for the treatment of cancer. These systems selectively deliver therapeutic agents to target tissues, cells and cell compartments and release their loads. Pharmacological properties, release profile, and therapeutic results are improved compared to the chemotherapeutic delivery as free drugs for cancer therapy [22].

In this work, we examined the karyotypic stability and gene expression of the descendants of Chinese hamster RJK cells sensitive and resistant to the MDR agent surviving after exposure to polycation PAA in sublethal dose. The data obtained in this study showed that PAA in high concentration was extremely toxic for RJK and RJKEB cells. The treatment of these cells with PAA in sublethal concentration led to multidirectional disorganization of the karyotype: aneu(poly)ploidy and occurrence of a large number of chromosomal aberrations, primarily breakdowns. It is known that chromatid breaks, chromosomal breakdowns, and polyploidy are common for the initial stage of oncogenesis process [23]. Genomic instability facilitates the risk of malignant transformation. It is known that any karyotype contains chromosomes with increased fragility [24,25]. Our many years of studies of RJK cells revealed that the most sensitive to various types of stress was the chromosome 2 [26,27]. Other chromosomes are involved in rearrangements more randomly. Genomic instability generated by random (non-clonal) chromosomal abnormalities is a key driver in genome evolution, including cancer progression [28,29]. Karyotypic changes that occur in RJK cells after PAA treatment are mostly random. An important role in the disorganization of the karyotype is played by aneu(poly)ploidy. It is known that the emergence of aneu(poly)ploidy is associated with disorders in the mechanisms of cell division [30]. Once it has arisen, it can lead to abnormal chromosome segregation in mitosis. Disturbances in the cell division program can be spontaneous or induced by a stressor factor. It was shown that increased instability of the cell genome is typical for cancer cells. It may be associated with the presence in the karyotype of both unspecific aneuploid chromosomes resulted from random events and specific ones that enhance the genome instability, thereby contributing to an increase in the cancer potential. There is evidence that aneu/polyploidy, increasing genome instability, contributes to the drug resistance [31] and, as a consequence cell adaptability and tumor recurrence [32,33].

PAA in sublethal concentration had a strong effect on the cell division of the analyzed cells. It disordered the regular chromosome separation in mitosis and altered their morphology. It is interesting to note that although chromosomal instability has long been considered to contribute to tumor development, recent studies have shown that chromosomal instability can either contribute to or suppress tumor development. The choice is determined by the level of chromosomal instability. A low level of chromosomal instability facilitates tumor progression, while a high level often promotes the death of cancer cells [34]. It was demonstrated that sublethal heat stress may trigger non-tumorigenic karyotypic instability due to homologous recombination deficiency and decrease of oncogene expression in progeny of heat shock survived mesenchymal stromal cells [35]. Karyotypic destabilization in RJK-PAA and RJKEB-PAA cells does not seem to be significant for its viability.

Increased karyotypic instability in transformed cell lines may be accompanied by alteration in genetic expression. In this work, while trying to study the effect of PAA on RJK and RJKEB cells, we focused on the expression of genes that are targets for cancer treatment. Since the object of our study was the RJKEB cell line with the MDR phenotype, we first analyzed the change in the expression of the mdr1 gene. Real-time PCR showed that basal expression of the mdr1 gene was 10 times higher in these cells than in the RJK line. It correlated with the presence of additional HSR genetic material on chromosome Z6 in RJKEB cells. RJK-PAA and RJKEB-PAA cells did not exhibit increased expression of mdr1. Enhanced MDR1 expression is typical for cells with “classic” MDR. By contrast, cells without overexpression of *mdr1* gene and multidrug resistance phenotype are named as atypical MDR cells. These cells have altered expression of topoisomerase, glutathione-S-transferases and cytochrome P450-dependent oxidases which affect the DNA topology by introducing single- or double-stranded breaks with subsequent restoration and play an important role in the processes of replication and transcription. We found that four passages after the cell exposure to PAA, the expression of the topo2-α gene decreased compared with untreated cells. Topo2-α is essential for various cellular processes, such as DNA replication, transcription, recombination, and chromosome separation. Topo2-α is the target of many antitumor drugs which can directly bind with topo2-α, stabilize topo2-α–DNA complex that promote the death of tumor cells. Thus, inhibition of topo2-α may be a supportive anticancer strategy. Modified activity of topo2-α and its disordered regulation may be accompanied with genetic instability [36]. It was found that expression of the topo2-α protein in larynx carcinoma was positively correlated with aneuploidy of human chromosome 17. Aberrant expression of topo2-α and aneuploidy of human chromosome 17 contributed to tumor development and its progression. It shows that targeting topo2-α may provide a treatment strategy for patients with laryngeal cancer [37]. Low expression of topo2-α increased survival of patients with non-small cell lung cancer treated with amrubicin, a topo2-α inhibitor [38]. Thus, a decreased expression of topo2-α in the RJK-PAA and RJKEB-PAA cells may be indicative for enhanced sensitivity of these cells to drugs.

*C-fos* is a proto-oncogene overexpressed in different types of cancer. We found that its expression was higher in RJK cells. After cell exposure to PAA, expression of *c-fos* was significantly increased in RJKEB but not RJK cells. This protooncogene is a member of the Fos family of transcription factors. It binds to c-Jun and form heterodimeric activator protein-1 (AP-1) complexes that transcriptionally regulate the expression of many genes [39]. It has recently been shown that suppression of AP-1 inhibits mdr1 expression and reduces drug resistance in gastric cancer cells [40]. There is evidence that overexpression of *c-fos* in head and neck squamous cell carcinoma enhances epithelial-mesenchymal transition (EMT) and the expression of cancer cell markers (Nanog, c-Myc, Sox2 and Notch1) [41]. However, it is believed that c-fos may also be involved in tumor growth suppression [42]. For example, c-fos inhibits the proliferation of tumor hepatocytes [43]. In ovarian cancer (OvCa) high c-fos expression correlates with a well differentiated phenotype [44]. It decreased with metastasis compared with primary tumors [45]. High c-fos expression inhibited metastasis in OvCa cells, altered cellular adhesive properties and is an independent favorable prognostic factor for OvCa cells [46]. C-fos binds to the miR-551 promoter in breast cancer cells, reduces cell adhesion, and blocks tumorigenesis [47].

HSP and heat shock factors (HSF) play a significant role in cancer progression. HSF1 is involved in the regulation of a specific P-pg transporter. It was shown that the P-pg1 promoter contains heat shock elements (HSE). Overexpression of HSF1 increased activity of p-glycoprotein and produced resistance to the chemotherapeutic agent doxorubicin [48]. Thus, HSF and HSP can be an important target for MDR reversibility [49,50]. Numerous studies have shown the relationship between membrane-associated HSP and drug resistance and the possibility to use HSP as biomarkers for tumor detection [51]. The results of our study showed that the basal level of expression of the *hsc70* and *grp78* genes was significantly higher in RJKEB cells with MDR phenotype. No significant difference in expression of *hsp90* was found in the RJK and RJKEB cells. After cell exposure to PAA the expression of *hsp90* and *hsc70* decreased. The level of *grp78* remained unchanged. High HSP level is common for various cancer cells. In cancer cells, HSP70 supports mitotic signals and suppresses apoptosis and aging caused by oncogenes [52]. It has been shown that enhanced expression of HSP70 is accompanied with activation of mesenchymal markers N-cadherin, MMP2, SNAIL, and vimentin and promotes metastasis of breast cancer [53]. Overexpression of HSC70 stimulates glioma cell proliferation, migration, and invasion through phosphorylation and activation of FAK, Src, and Pyk2 [54]. GRP78, a resident protein in the endoplasmic reticulum (ER), is also overexpressed in various tumor cells. It promotes cancer cell survival by preventing autophagy and apoptosis associated with ER stress [55]. HSP70 can translocate into the plasma membrane of cancer cells and enter the intercellular space, where it mediates antitumor immune responses [56]. HSP90 is the most examined member of the HSP family due to its many functions in cancer development. This protein is often overexpressed and is associated with poor prognosis for multiple tumors, including cholangiocarcinoma, lung cancer, stomach and breast cancer, and glioblastoma [57]. Increased expression of HSP90 activates oncogenic protein kinases JAK2/STAT3, PI3K/AKT and MAPK that facilitates cancer cell progression cancer [58]. It has been demonstrated that HSP90 interacts with the promoter of human telomerase reverse transcriptase (hTERT). Its expression is often enhanced during cell immortalization [59]. Elevated levels of HSP90 have been found in breast and prostate cancer [60,61]. High expression of the HSP70 family (*hsc70* and *grp78*) in the RJKEB cell line corresponds to enhanced expression of chaperones in cancer cells with the MDR phenotype. In RJK and RJKEB cells surviving sublethal exposure to PAA, a decreased level of *hsp90* and *hsc70* may indicate their reduced resistance to drugs.

Increased expression of *c-fos* in the RJKEB cells and decreased expression of *topo2-α* and HSP genes (*hsp90* and *hsc70*) point to altered cell sensitivity to MDR agents. To verify this assumption, we tested the resistance of cells of both lines surviving after exposure to PAA to DOX. It was found that DOX resistance of RJKEB-PAA and parental RJKEB cells did not differ. It shows that the progeny of RJKEB after PAA exposure cells retained MDR phenotype.

Cell viability in our experiments was evaluated with MTT assay which reflects the metabolic activity of mitochondria. Differences in the proliferation rate of different cells can affect the cytotoxicity index. A reliable indicator of proliferating cells survival is their ability to multiply, which is determined by cell cloning. It was found that it was impossible to clone RJK and their progeny RJK-PAA cells in DOX presence. The cloning efficiency of the RJKEB cells after DOX treatment markedly declined compared to RJKEB cells. It is known that the main targets of DOX in a cell are topo2-α and antioxidant defense enzymes [62]. Their interaction disturbs DNA replication, produces DNA breaks, and alters cellular redox status that results in cell cycle arrest and apoptosis. RJKEB cells exhibit high level of *mdr1* expression which was not altered after PAA exposure. However, expression of *topo2-α* and *HSP* in RJKEB-PAA cells was decreased compared with parental cells. Probably, the low cloning efficiency of RJKEB-PAA cells after DOX treatment indicates that the population contains a large number of cells with injuries that impede their clonal propagation. These findings suppose that PAA treatment enhanced the sensitivity of RJKEB cells to drugs. The mechanism of polycation effect on the cell genome is still unknown. However, there is evidence of a cytotoxic effect of polycation poly(ethylenimine) associated with a disturbed membrane potential of mitochondria and apoptosis [63]. Using proteomic analysis, it was shown that polycations interact in the cell with HSP and some other proteins involved in apoptosis induction in tumor cells [64]. In our work, increased DOX cytotoxicity was observed in RJK-PAA and RJKEB-PAA cells. Supposedly, it was associated with decreased expression of *HSP*, *topo2-α* and increased expression of *c-fos*. Our results are consistent with data from Roy et al. [65] that PAA can enhance the effect of doxorubicin in the intracellular delivery of this drug to tumor cells.

## 5. Conclusions

In the present work, we demonstrated that treatment of Chinese hamster immortalized RJK and RJKEB cells with cytotoxic doses of PAA selected cells with increased karyotypic instability were accompanied by changes in the expression of *p53*, *c-fos*, *topo2-α*, *hsp90*, *hsc70* genes and did not contribute to MDR progression. Enhanced expression of tumor suppressor *p53* and declined expression of *HSP* and *topo2-α* point to a decrease in their resistance to drugs. This suggestion was confirmed by the increased sensitivity of these cells to the toxic effects of DOX. Thus, we can conclude that PAA did not facilitate and even slightly reduced the cell tumorigenic potential and thus can be used for intracellular drug delivery, for anticancer therapy, in particular.

## Figures and Tables

**Table 1:** The primers and conditions for qRT-PCR.

Symbol	Primer Sequence	Amplification Conditions	PCR Product Size (bp)	NCBI Reference Sequence
p53	F 5′ GTTGGCTCTGACTGTACCAC 3′ R 5′ AGGGTGAAATATTCTCCATC 3′	93 °C, 20 s, 57 °C, 20 s, 72 °C 30 s	317	NM_001243976.1
topo2-α	F 5′ GGGAGACTCAGCCAAAACAC 3′ R 5′ CAGCATCATCTTCAGGTCCA 3′	93 °C, 20 s, 58 °C, 20 s, 72 °C 30 s	560	NM_001246738.1
mdr1	F 5′ ATCGACGGTCAGGACATCAG 3′ R 5′ TTCAGCGATAGTGGTGGCAA 3′	93 °C, 20 s, 60 °C, 20 s, 72 °C 30 s	102	XM_027439202.1
c-fos	F 5′ GCAGCCAAATGCTGGAATCG 3′ R 5′ CCAGTGATGTCTTGGGCTCA 3′	93 °C, 20 s, 60 °C, 20 s, 72 °C 30 s	310	NM_001246683.1
grp78	F 5′ GATGCGGCCAAGAACCAGCT 3′ R 5′ CGCATGACATTCAGTCCAGC 3′	93 °C, 20 s, 63 °C, 20 s, 72 °C 30 s	359	NM_001246739.2
hsp90	F 5′ AATCGGAAGAAGCTTTCAGA 3′ R 5′ GTGCTTGTGACAATACAGCA 3′	93 °C, 20 s,56 °C, 20 s, 72 °C 30 s	257	NM_001246821.1
hsc70	F 5′ ATCCCCAAGATTCAGAAGCT 3′ R 5′ TTGATGAGGACAGTCATGAC 3′	93 °C, 20 s, 56 °C, 20 s, 72 °C 30 s	218	NM_001246729.1
actin	F 5′ GCTGAGAGGGAAATTGTGCGTG 3′ R 5′ CGGTGGACGATGGAGGGGCCG 3′	93 °C, 20 s, 68 °C, 20 s, 72 °C 30 s	506	XM_007648665.3

The primers and conditions for qRT-PCR. Abbreviations: qRT-PCR—quantitative reverse transcription polymerase chain reaction; F—forward; R—reverse; p53—tumor protein p53 (Tp53); topo 2-α—DNA topoisomerase 2; mdr1—multidrug resistance 1; ATP binding cassette subfamily B member 1 (Abcb1); c-fos—Fos proto-oncogene; grp78—heat shock protein family A (Hsp70) member 5; hsp90—heat shock protein 90 alpha family class A member 1 (Hsp90aa1); hsc70—heat shock protein family A (Hsp70) member 8 (Hspa8); actin—actin beta (Actb).

**Table 2:** Karyotype characterization of RJK, RJKEB, RJK-PAA, RJKEB-PAA cells.

			Variability of Chromosome Copy Number	Cell Number with Modified Chromosome Structure, %			
Cells	Chromosome Variability	Modal Class		Breaks with Preservation of Genet Material	Deletions	Translocations	HSRs Presence
RJK	16-23	17-19	0-2	16	22	11	0
RJK-PAA	15-32	Not evident	0-5	11	50	17	0
RJKEB	18-32	18	0-3	5	20	10	100
RJKEB-PAA	15-21	18-20	0-3	33	55	0	100

Karyotype characterization of RJK, RJKEB, RJK-PAA, RJKEB-PAA cells. Abbreviations: RJK—Chinese hamster cells CHLV-79 RJK; RJKEB—RJK resistant to ethidium bromide; PAA—polyallylamine hydrochloride; RJK-PAA—RJK cells after PAA treatment; RJKEB-PAA—RJKEB cells after PAA treatment; EB—ethidium bromide; HSR—homogeneously stained region.
